# A novel mutation in *ST14* at a functionally significant amino acid residue expands the spectrum of ichthyosis-hypotrichosis syndrome

**DOI:** 10.1186/s13023-017-0728-8

**Published:** 2017-12-06

**Authors:** Leila Youssefian, Andrew Touati, Amir Hossein Saeidian, Omid Zargari, Sirous Zeinali, Hassan Vahidnezhad, Jouni Uitto

**Affiliations:** 1 Department of Dermatology and Cutaneous Biology, Sidney Kimmel Medical College, Thomas Jefferson University, 233 S. 10th Street, Suite 450 BLSB, Philadelphia, PA 19107 USA; 2 Department of Medical Genetics, School of Medicine, Tehran University of Medical Sciences, Tehran, Iran; 3 Drexel University College of Medicine, Philadelphia, PA USA; 4 Dana Clinic, Rasht, Iran; 5 Molecular Medicine Department, Biotechnology Research Center, Pasteur Institute of Iran, Tehran, Iran; 6 Kawsar Human Genetics Research Center, Tehran, Iran

**Keywords:** Ichthyosis, Hypotrichosis, Ichthyosis-hypotrichosis syndrome, *ST14*, Matriptase, Next-generation sequencing

## Abstract

**Background:**

Mutations in the *ST14* gene, encoding the serine protease matriptase, have been associated with ichthyosis-hypotrichosis syndrome (IHS), a Mendelian disorder with skin and hair manifestations which include, in addition to ichthyosis and hypotrichosis, hypohidrosis and follicular atrophoderma. However, the understanding of the specific consequences of mutations in *ST14* on the development of this syndrome is incomplete.

**Results:**

Using a targeted next-generation sequencing array of 38 ichthyosis-associated genes on a large cohort of 180 ichthyosis patients from a primarily consanguineous background, a previously unreported homozygous p.Asp482Asn mutation in *ST14* was identified in a patient with IHS. This mutation affects an essential site within a ligand-binding domain of matriptase. Comparison with previous reports of IHS allowed further delineation of the phenotype of IHS in correlation with mutations present in these patients. Histological and ultrastructural analysis of skin and hair identified novel features in this disorder.

**Conclusions:**

This study correlates genotypic and phenotypic features of the rare disorder, IHS, expands the spectrum of pathology associated with the disorder, and provides clinical evidence of the importance of the Asp482 amino acid, previously shown to have an essential role in matriptase activation.

## Background

The inherited ichthyoses in the spectrum of Mendelian disorders of cornification affect the generalized integument with hyperkeratosis and scaling [1]. The inherited forms of ichthyosis have been classified as either nonsyndromic or syndromic, with nonsyndromic forms having phenotypic manifestations exclusively in the skin, and syndromic ichthyoses involving additional structures and organs. Several syndromic forms of ichthyosis include primary hair pathology, such as Netherton syndrome (NS), neonatal ichthyosis-sclerosing cholangitis syndrome (NISCH), and others (Table 1). In this category, an ichthyotic syndrome, with mild to moderate ichthyosis and multiple abnormalities in hair, including hypotrichosis, was first reported in 1998 [2]. Since then, multiple additional cases have been reported, and this syndrome has gained the name of ichthyosis-hypotrichosis syndrome (IHS; OMIM# 602400).

**Table 1:** Syndromic forms of ichthyosis with hair pathology

Syndrome	Gene	Protein function	Inheritance	Hair involvement	OMIM#
Ichthyosis-hypotrichosis syndrome	*ST14*	Matriptase-prostasin cascade activation	AR	Hypotrichosis, lightly colored hair, follicular atrophoderma	602,400
Netherton syndrome	*SPINK5*	Matriptase-prostasin cascade inhibition	AR	Sparse, brittle hair, trichorrhexis invaginata	256,500
NISCH syndrome	*CLDN1*	Tight junction formation	AR	Hypotrichosis, alopecia	607,626
IFAP syndrome	*MBTPS2*	Regulation of sterol synthesis	XLR	Generalized atrichia	308,205
Trichothiodystrophy	*ERCC2* *ERCC3* *GTF2H5* *MPLKIP* *GTF2E2*	Transcription factor activation, nucleotide excision repair	AR	Thin, brittle hair, alternating light and dark bands under polarized light	601,675 616,390 616,395 234,050 616,943
Conradi-Hünermann-Happle syndrome	*EBP*	Sterol biosynthesis	XLD	Sparse hair, cicatricial alopecia, follicular atrophoderma	302,960
KID syndrome	*GJB2*	Gap junction formation	AD	Alopecia totalis	148,210

*Abbreviations*: *NISCH* Neonatal Ichthyosis-Sclerosing Cholangitis, *IFAP* Ichthyosis Follicularis, Atrichia, and Photophobia, *KID* Keratitis-Ichthyosis-Deafness, *AR* Autosomal Recessive, *XLR* X-linked Recessive, *XLD* X-linked Dominant, *AD* Autosomal Dominant

The phenotype of IHS exists in a spectrum. The manifestations of ichthyosis are typically mild to moderate, but have been reported as severe in one case [2]. Universal hypotrichosis occurs in all patients, characterized by curly, relatively lightly pigmented, short, sparse hair. A childhood receding frontal hairline and loss of the lateral portions of the eyebrows are specific features identified in multiple cases. A more severe presentation of IHS has been associated with the findings of hypohidrosis and follicular atrophoderma, i.e.*,* ice pick-like depressions in the place of follicular units [2–4]. This constellation of findings has been referred to as IFAH (ichthyosis and follicular atrophoderma with hypotrichosis and hypohidrosis), contrasting the more mild presentations of IHS which have been referred to as ARIH (autosomal recessive ichthyosis with hypotrichosis) [5]. Additional findings in IHS include corneal opacities, photophobia [5], pinguecula [6], and pitted and conical incisors [4], although their association with the syndrome is not well-defined.

Biallelic mutations in *ST14*, encoding the serine protease matriptase, have been reported in IHS since 2007, and up to date, six distinct mutations in this gene have been associated with IHS [4–8]. Here, we report a case of IHS caused by a previously unreported homozygous missense mutation in *ST14* at a functionally significant site of the protein, with a comprehensive clinical analysis, thus providing improved characterization of the genotypic and phenotypic features of IHS.

## Methods

### Patient recruitment

This study was approved by the institutional review board of the Pasteur Institute of Iran, and all subjects and the parents of minors gave written informed consent to participate in research and to publish their images. In this study, 180 extended families affected by nonsyndromic and syndromic forms of ichthyosis, diagnosed in various medical centers in Iran, were evaluated.

### Next-generation sequencing array

DNA was extracted from peripheral blood samples by salting-out method. Target enrichment was performed using the TruSeq Custom Amplicon kit (Illumina, San Diego). DesignStudio (Illumina) was used for library design. All coding exons, at least 20 bp of the intron at each intron-exon boundary, and up to 50 bp of 5′- and 3′-untranslated regions were targeted. The designed library contained 38 genes (*ABCA12, ABHD5, AGPS, ALDH3A2, ALOX12B, ALOXE3, AP1S1, ARSE, CERS3, CLDN1, CYP4F22, EBP, ELOVL4, GJB2, GJB3, GJB4, GJB6, KRT1, KRT10, KRT2, KRT9, LIPN, LOR, NIPAL4, PEX7, PHYH, PNPLA1, PNPLA2, POMP, SLC27A4, SNAP29, SPINK5, ST14, STS, TGM1, TGM5, VPS33B,* and *ZMPSTE24*), divided into 351 targets covered by 558 amplicon probes, which were designed to cover 99% of targeted bases. A total of 93.2% of the reads were aligned to the human genome, with the mean coverage of the target region being 493×. In addition, only 0.4% of bases of the target region were not covered by any sequence read, indicating that 99.6% of all target region bases were sequenced at least once.

Reads were analyzed and aligned as previously described [9]. Variants were classified according to the American College of Medical Genetics and Genomics guidelines [10], population variant frequencies were generated from the Exome Aggregation Consortium database (ExAC.broadinstitute.org), and predictive softwares were used to analyze the consequences of missense mutations including MutationTaster (mutationtaster.org), Sift v.1.03 (sift.jcvi.org), PROVEAN (provean.jcui.org), and PolyPhen-2 (genetics.bwh.harvard.edu).

### Polymerase chain reaction (PCR) and Sanger sequencing

PCR was performed using Taq polymerase (Qiagen, Valencia, CA) according to the manufacturer’s instructions, with custom-designed primers. The PCR products were bidirectionally sequenced using an automated sequencer (3730; Applied Biosystems, Foster City, CA). Mutation positions are reported in reference to NM_021978.

### Scanning electron microscopy

Hair samples obtained from the scalp of the proband were mounted on scanning electron microscopy sample holders, sputter coated with gold/palladium alloy, and imaged using a FEI FEG Quanta 250 (FEI, Hillsboro, Oregon) operated at 5 kV. Multiple hairs were examined, and consistent findings were noted.

## Results

### Case report

**Fig. 1:**
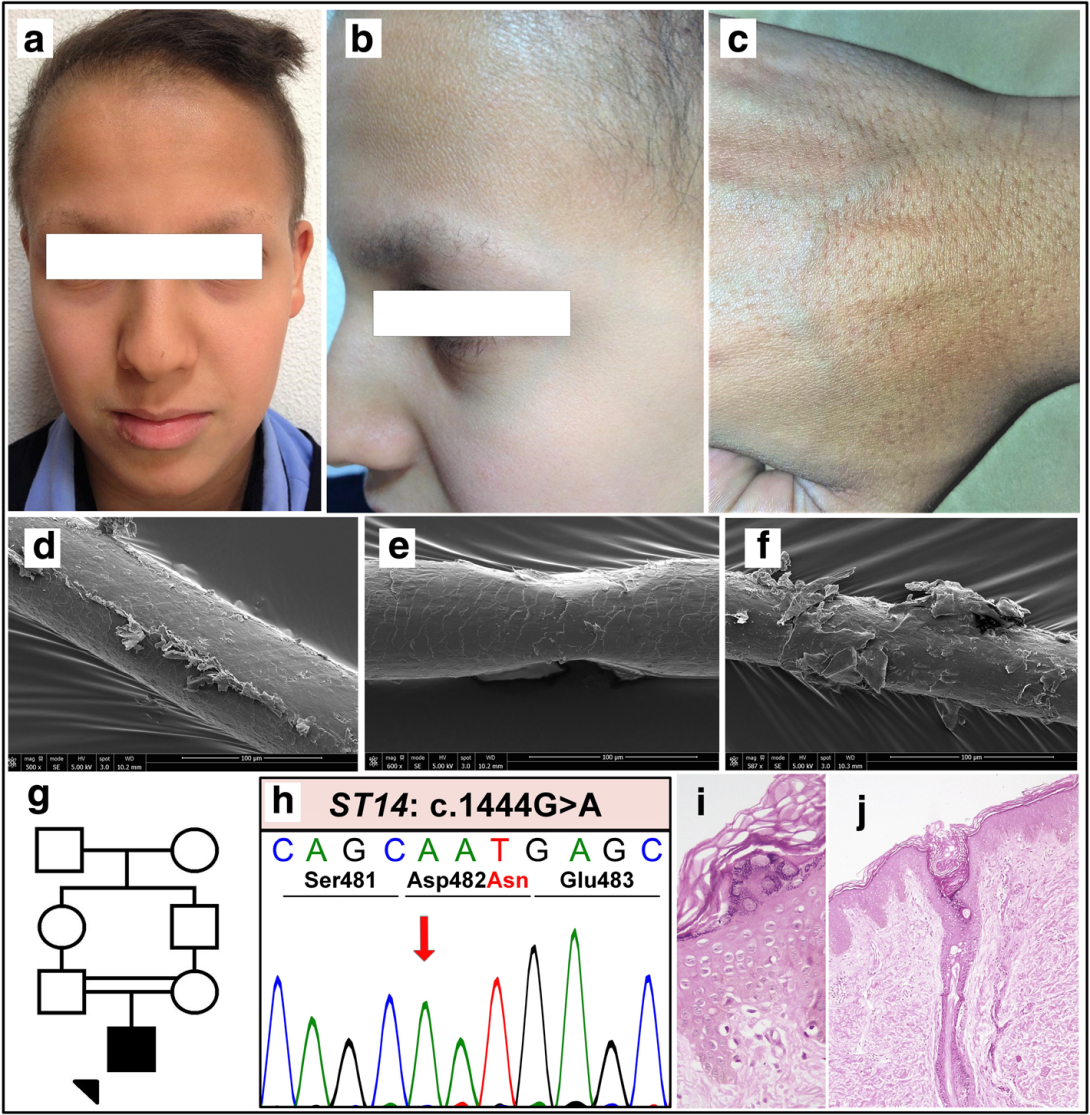
Clinical features of the index patient following initiation of acitretin therapy, including hypotrichosis and ichthyosis (**a**-**b**), along with follicular atrophoderma (**c**). Scanning electron microscopy of the patient’s scalp hair displaying longitudinal ridging (**d**), pseudomonilethrix (**e**), and cuticular fraying (**f**). Pedigree showing first-cousin consanguinity in the patient’s parents (**g**). Sanger sequencing of the index patient’s genomic DNA displaying the homozygous p.Asp482Asn mutation in *ST14* (**h**). Skin histology of the patient’s epidermis (**i**) and a hair follicle (**j**)

The index case, an Iranian male of Persian decent, was born to consanguineous parents (Fig. 1). No collodion membrane was noted. Skin findings were present at birth including a moderate ichthyosis consisting of fine white scales affecting the entire body, but sparing the palms and soles. Total alopecia was noted in infancy, but in childhood, he developed short, coarse scalp hair of a light brown color as compared to the black hair present in the rest of his family (Fig. 1a and b). When examined at the age of 16 years, mild regression of the frontal hairline and sparseness of the hair, including scalp and body hair, as well as lateral eyebrows were noted, however, eyelashes appeared normal (Fig. 1a and b). Follicular atrophoderma was present on the dorsal hands and forearms (Fig. 1c). There was no evidence of hypohidrosis, ocular or dental abnormalities. The palms and soles were clear, and specifically, there was no palmoplantar keratoderma. The skin findings had gradually improved over the years, and the ichthyosis responded well to acitretin therapy initiated four years earlier, initially 10 mg/day and more recently 25 mg/day.

Hematoxylin and eosin staining of a 4 mm punch biopsy from flank skin revealed irregular acanthosis, mild basal layer hyperpigmentation, diminished granular layer, and mild basket weave hyperkeratosis extending focally to hair follicles associated with keratotic follicular plugging (Fig. 1i and j). Scanning electron microscopy of hair revealed significant pathology, including longitudinal ridging and grooving, monilethrix-like tapered restrictions, and cuticular fraying (Fig. 1d-f).

### Genetic diagnosis

A cohort of 180 patients with a clinical diagnosis of ichthyosis excluding ichthyosis vulgaris was analyzed with a customized next-generation sequencing (NGS) array targeting 38 ichthyosis-associated genes (for the list of genes, see Methods). Among these patients tested, the proband in this case was found to have a homozygous missense mutation, c.1444G > A (p.Asp482Asn), in *ST14* by using the NGS array, and the results were confirmed with bi-directional Sanger sequencing (Fig. 1h). Unfortunately, the DNA of the parents was not available to confirm the familial segregation of the mutant alleles. The mutation occurred at a highly conserved residue and was predicted to be damaging by multiple computational prediction softwares (see Methods). The mutation was absent in a control population of 60,706 individuals in the ExAC database, which excludes individuals affected with severe pediatric diseases, providing evidence for the pathogenicity of this *ST14* mutation.

## Discussion

### Clinical comparison to previous reports of IHS

Ichthyosis-hypotrichosis syndrome is a rare disease with broad implications in skin and hair function. Here, we report an additional IHS case with a previously unreported homozygous missense mutation in *ST14*, expanding the genotypic spectrum of this syndrome. In likeness to the clinical presentation of previous IHS cases, our patient had ichthyosis and typical hair findings (Table 2). Interestingly, follicular atrophoderma was present in the absence of hypohidrosis. This is consistent with the notion that IFAH and ARIH are representative of degrees of severity of IHS, with follicular atrophoderma and hypohidrosis as possible features, but not necessarily occurring in combination. Our patient lacked any ocular or dental findings, which have been reported in association with several other *ST14* mutations (Table 2). The association of IHS with previously reported ocular and dental findings remains unclear, especially given that no such findings were present in our case. However, *St14*-hypomorphic mice have been shown to have findings of enamel dysgenesis on scanning electron microscopy [11], suggesting that IHS patients should be carefully examined for dental anomalies.

**Table 2:** Clinical features of ichthyosis-hypotrichosis syndrome with associated mutations in *ST14*

Mutation	c.3G > A	c.598 + 1G > A	c.1444G > A (p.D482N)	c.1558G > C (p.E520Q)	c.2034delG (p.L678Ffs*84)	c.2269 + 1G > A	c.24791G > A (p.G827R)
Mutation type	Start codon	Splice site	Missense	Missense	Frameshift	Splice site	Missense
Reference	[7]	[8]	Current report	[4]	[6]	[6]	[5]
Description of ichthyosis	Generalized scaling, sparing flexures	Generalized erythema and scaling, later localized to back	Fine white generalized scale	Fine gray scale sparing elbows and knees	Generalized scaling, sparing flexures and face	Generalized scaling, sparing elbows, knees, and face	Gray to brown generalized scaling, sparing face
Description of hypotrichosis	Light brown, coarse, curly scalp hair, generalized sparse hair including eyebrows, eyelashes	Light brown, receding frontal hairline, partial loss of eyebrows, curled eyelashes	Light brown, coarse scalp hair, recession of frontal hairline, generalized sparse hair	Coarse scalp hair, generalized sparse hair including eyebrows and eyelashes	Light brown, woolly hair, recession of frontal hairline	Sparse scalp, eyebrow, and body hair, with recession of frontal hairline	Curly, light. Sparse, generalized hair
Palmoplantar keratoderma	–	+	–	–	–	–	–
Ocular anomalies	Blepharitis	–	–	–	–	Pinguecula, corneal opacities	Corneal opacities
Dental anomalies	–	–	–	Conical teeth, pitting	–	–	Conical teeth, pitting
Follicular atrophoderma	–	–	+	+	+	+	–
Hypohidrosis	–	–	–	–	+	+	–

Skin histology was consistent with previous reports, including acanthosis, hypogranulosis, and basket weave orthohyperkeratosis [3, 6], although keratotic follicular plugging, present in our patient, has not been previously noted. The patient in this report started retinoid therapy prior to the performance of his skin biopsy, which may account for the relatively mild degree of hyperkeratosis seen in the stained section.

Scanning electron microscopy of hair has been performed in two of the previous cases of IHS, with findings of subtlety of the hair grooves, increased irregularity of the cuticular scale edges [7], and pili bifurcati [5]. The additional findings of pseudomonilethrix and longitudinal ridging in this case are of unclear clinical relevance, given the similar gross hair pathology in other IHS cases, but highlight the fact that the specific hair defects due to matriptase dysfunction remain poorly understood.

### Molecular pathology of the *ST14* gene in IHS

**Fig. 2:**
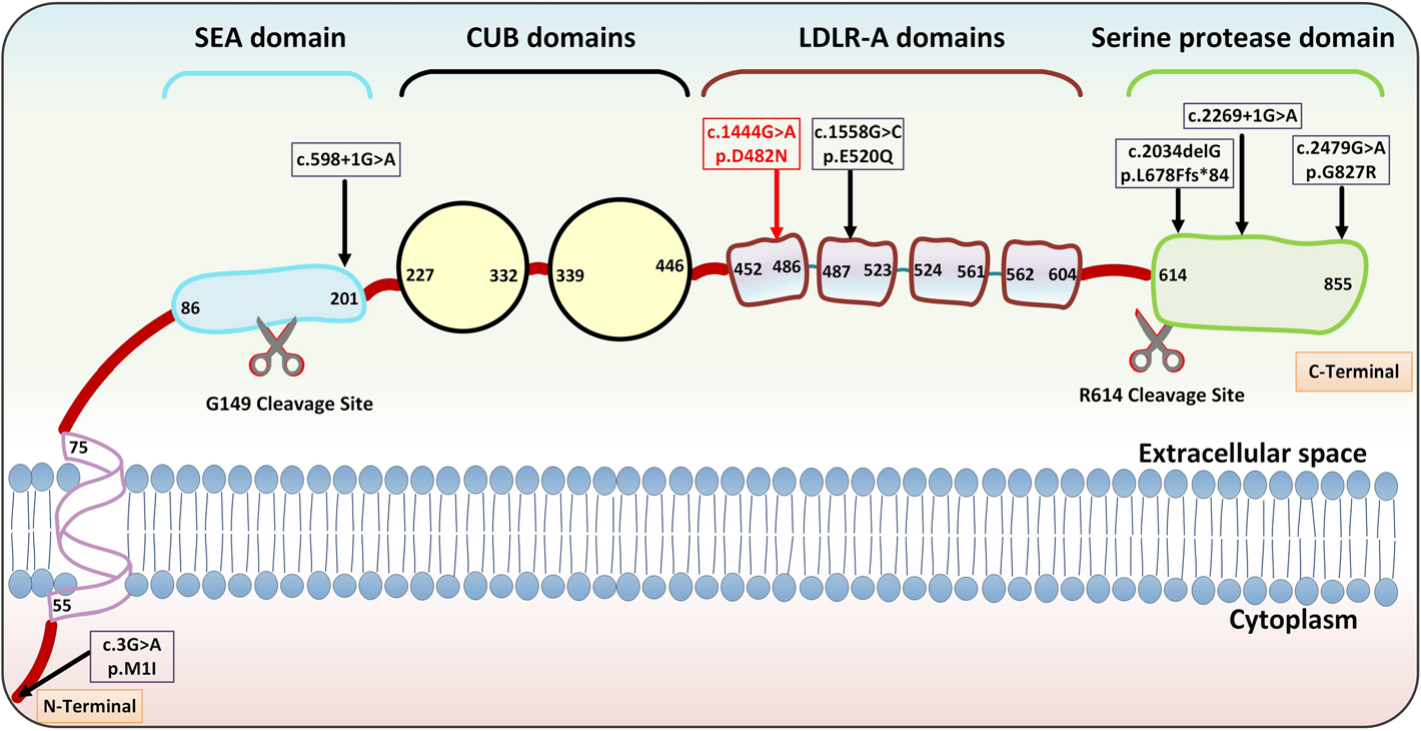
Representation of the matriptase transmembrane protein product, with domains labeled with their first and last amino acids. Sites of cleavage required for matriptase activation are shown below the domains. Mutations in the *ST14* gene causing IHS are displayed at their corresponding sites of effect on matriptase at the amino acid level (boxed). The currently reported mutation is exhibited in red at the first LDLR-A domain. SEA, sea urchin sperm protein-enteropeptidase-agrin; CUB, C1r/C1s, urchin embryonic growth factor bone morphogenic protein; LDLRA; low density lipoprotein receptor class A

The matriptase protein encoded by the *ST14* gene consists of several major domains, including (a) an N-terminal transmembrane domain, (b) a sea urchin sperm protein-enteropeptidase-agrin (SEA) domain containing a cleavage site required for proteolytic activation of matriptase, (c) two adjacent C1r/C1s-urchin embryonic growth factor bone morphogenic protein (CUB) domains required for efficient N-terminal proteolytic processing, (d) four low density lipoprotein-receptor class A (LDLR-A) domains in tandem, and (e) the catalytic domain which encodes the serine protease portion of matriptase [12] (Fig. 2). Previously, six distinct mutations have been reported in *ST14* in association with IHS. One abrogates the start codon, two are missense mutations, one is a frameshift deletion, and two are canonical splice site mutations (Table 2). Our patient’s mutation adds an additional missense mutation to this group. Up to this point, genotype-phenotype correlations have not been clearly developed in IHS in terms of overall disease severity. In previous cases as well as our own, no clear trend can be ascertained between the severity of ichthyosis or hypotrichosis and mutation type. Thus, we examined which mutations have been associated with follicular atrophoderma and hypohidrosis, features of what was previously termed IFAH and is now considered as the more severe end of the spectrum of IHS. Cases with follicular atrophoderma were associated with a canonical splice site mutation (c.2269 + 1G > A) and an indel mutation (c.2034delG) [6], p.Glu520Gln (note: erroneously reported as Glu519Gln) [4],as well as the p.Asp482Asn mutation in our case. These latter two mutations occur on highly conserved sites on the LDLR-A domains (Fig. 2). LDLR-A domains are present on the *LDLR* gene, as well as in multiple type II transmembrane serine proteases, and a series of negatively charged amino acids including aspartic acid and glutamic acid residues at the C-terminal end of each domain forms a region theorized to be important for ligand binding [13]. Remarkably, induced point mutations at the Asp482 and Asp519 codons cloned into a human cell model have been shown to drastically reduce matriptase activation, providing strong evidence for the loss of matriptase activity in our patient [12]. The specific functions of the LDLR-A domains of matriptase remain unknown, although these findings raise the possibility that dysfunction of these domains may particularly contribute to the development of follicular atrophoderma as compared to the clinical consequences of mutations in other sites on *ST14*. The presence of follicular atrophoderma in association with a frameshift mutation and canonical splice site mutation indicate that a total loss of function of matriptase may also result in this clinical feature, although it is unclear why this is not the case in all expected loss-of-function mutations. Hypohidrosis, absent in our case, has only been reported in association with two mutations: a canonical splice site mutation (c.2269 + 1G > A) [6] and the frameshift deletion (c.2034delG) [3] (Table 2). The absence of hypohidrosis in our case as well as in the two previously reported missense mutations suggests that only severe loss-of-function of matriptase can result in cutaneous abnormalities significant enough to lead to hypohidrosis.

## Conclusion

We report a case of IHS with a homozygous, previously unreported missense mutation in *ST14*. This is the seventh mutation associated with IHS, with a unique combination of clinical and ultrastructural findings. This report provides corroborative evidence that the LDLR-A domains of *ST14* are essential for matriptase function, particularly at its highly conserved aspartic acid residues.

## Abbreviations

ARIH: Autosomal recessive ichthyosis with hypotrichosis
CUB: C1r/C1s-urchin embryonic growth factor bone morphogenic protein
IFAH: Ichthyosis and follicular atrophoderma with hypotrichosis and hypohidrosis
IHS: Ichthyosis-hypotrichosis syndrome
LDLR-A: Low density lipoprotein-receptor class A
NGS: Next-generation sequencing
NISCH: Neonatal ichthyosis-sclerosing cholangitis syndrome
NS: Netherton syndrome
PCR: Polymerase chain reaction
SEA: Sea urchin sperm protein-enteropeptidase-agrin
